# Lateral lymph node dissection combined with endoscopic submucosal dissection for early rectal cancer complicated with ulcerative colitis: A case report

**DOI:** 10.1097/MD.0000000000043160

**Published:** 2025-07-04

**Authors:** Baosu Huang, Bing Zhu, RunZhi Liu, Bin Ren

**Affiliations:** a Affiliated Hospital of Shandong Second Medical University, School of Clinical Medicine, Shandong Second Medical University, Weifang, Shandong, China; b School of Clinical Medicine, Shandong Second Medical University, Weifang, Shandong, China.

**Keywords:** combined laparoscopic-endoscopic technique, Early rectal cancer, endoscopic submucosal dissection, lateral lymph node dissection, ulcerative colitis

## Abstract

**Rationale:**

Early rectal cancer is mostly confined to the mucosa or submucosa, and metastasis to lymph nodes is rare. Stage 0 rectal cancer can usually be cured by endoscopic resection. However, when patients present with enlarged lateral lymph nodes of uncertain nature, additional surgical intervention should be considered. This article reports a case of early rectal cancer combined with ulcerative colitis. Preoperative imaging examination revealed enlarged right-sided group 263D lymph nodes. Since the nature of the enlarged lymph nodes cannot be determined, it was decided to perform lateral lymph node dissection combined with endoscopic submucosal dissection to achieve clear diagnosis and treatment. This article aims to illustrate the importance of individualized treatment for patients with early rectal cancer with complex clinical backgrounds and the clinical value of lateral lymph node dissection combined with endoscopic submucosal dissection.

**Patient concerns:**

The patient was a 66-year-old male who presented with blood in the stool for 1 month with no family history of hereditary tumors.

**Diagnosis:**

Colonoscopy and further biopsy diagnosed early rectal malignancy (stage 0). Pelvic magnetic resonance and enhanced computed tomography showed enlarged right group 263D lymph nodes with well-defined borders, approximately 1.2 cm in diameter. Together with the patient’s history, the possibility of metastasis of rectal cancer was considered, but the lymph nodes were diagnosed as reactive hyperplasia after lateral lymph node dissection.

**Interventions:**

Since the nature of the enlarged lymph nodes could not be determined, it was decided to first perform lateral lymph node dissection to rule out the possibility of metastasis, followed by endoscopic submucosal dissection to remove the tumor.

**Outcomes:**

The patient recovered well after surgery. Pathological examination showed reactive hyperplasia of the lymph nodes, and the tumor was completely resected with negative margins.

**Lessons:**

Lymph node metastases rarely occur in early rectal cancer, but this possibility should not be ignored, especially when dealing with patients with complex clinical backgrounds. This case also emphasizes the importance of individualized diagnosis as well as the extremely high clinical value of Lateral lymph node dissection combined with endoscopic submucosal dissection, and provides a reference for the diagnosis and treatment of similar cases.

## 1. Introduction

Ulcerative colitis (UC) is an idiopathic chronic inflammatory disease of the colon mucosa that originates in the rectum and spreading proximally. Most of them spread continuously to the entire or part of the colon.^[1]^ It is also one of the risk factors for rectal cancer. The incidence of rectal cancer is closely related to its course and severity. Early rectal cancer (ERC) is usually confined to the mucosa or submucosa, and lymph node metastasis rarely occurs. The incidence of lateral lymph node metastasis is only 0.5% to 0.9%.^[2]^ Stage 0 ERC can usually be treated by endoscopic resection. When the tumor is large, it is especially suitable for endoscopic mucosal dissection (ESD) to remove the tumor for radical treatment, and ESD has become an important treatment for stage 0 and stage 1 rectal cancer.^[3]^ However, when lymph node enlargement occurs and its nature is unclear, additional surgical intervention must be considered.

This article reports a case of ERC combined with UC. As preoperative imaging examination revealed the presence and uncertain nature of enlarged right-sided group 263D lymph nodes, the patient was finally treated with the strategy of lateral lymph node dissection (LLND) combined with ESD. The purpose is to emphasize the importance of individualized diagnosis and treatment and the advantages of combined laparoscopic-endoscopic technique, and to explore the clinical value of LLND combined with ESD.

## 2. Case report

**Figure 1.**
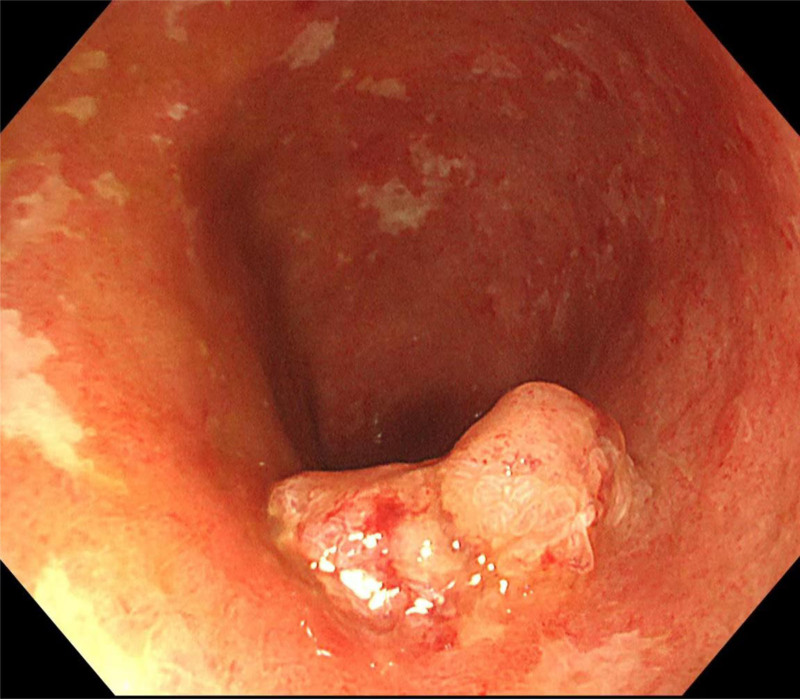
Colonoscopy revealed ulcerative colitis with a rectal mucosal mass about 2 cm in diameter.

**Figure 2.**
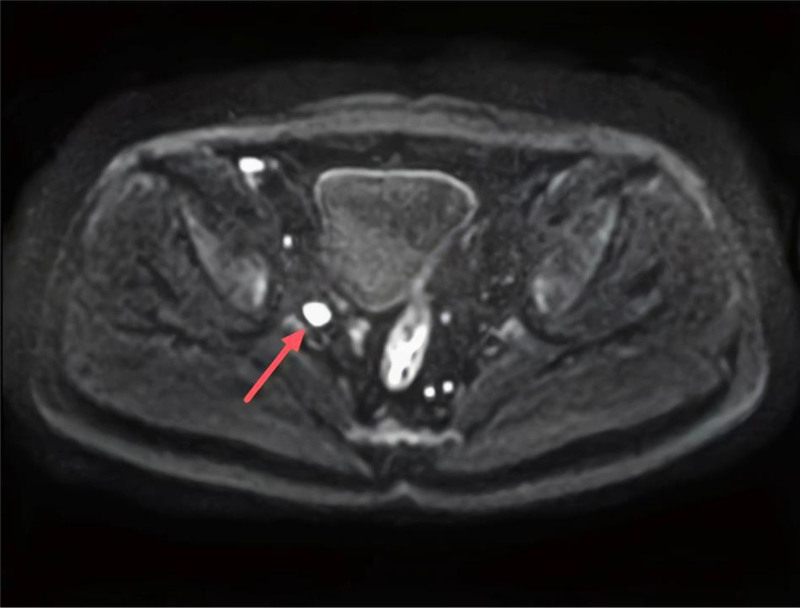
Pelvic MR showed the presence of enlarged lymph nodes of group 263D in the right side, with clear borders and a diameter of about 1.2 cm (red arrow). MR = magnetic resonance.

The patient was a 66-year-old male who presented with blood in the stool for 1 month with no family history of hereditary tumors. Colonoscopy revealed a rectal mucosal mass and UC (Fig. 1). Further biopsy and pathological diagnosis showed rectal villous adenoma with high-grade intraepithelial neoplasia, and focal canceration (intramucosal adenocarcinoma). Early rectal malignant tumor (stage 0) was diagnosed. Pelvic magnetic resonance and enhanced computed tomography showed enlarged right-sided group 263D lymph nodes, with clear boundaries and a diameter of about 1.2 cm (Fig. 2). The possibility of rectal cancer metastasis was considered in conjunction with the patient’s medical history. CEA, CA125 and CA19-9 were all within normal ranges.

Preoperatively, the patient was diagnosed with UC complicated with rectal malignant tumor. Considering that the patient’s rectal malignant tumor was stage 0, ESD treatment could be performed. However, due to the presence of enlarged lymph nodes in the right lateral group 263D and the inability to determine the nature of the enlarged lymph nodes, it was decided to carry out LLND to clarify the nature of the lymph node, and then ESD or radical resection of rectal cancer was performed according to the nature of the lymph node. The patient’s enlarged lymph node was reactive hyperplasia, so ESD treatment was decided.

**Figure 3.**
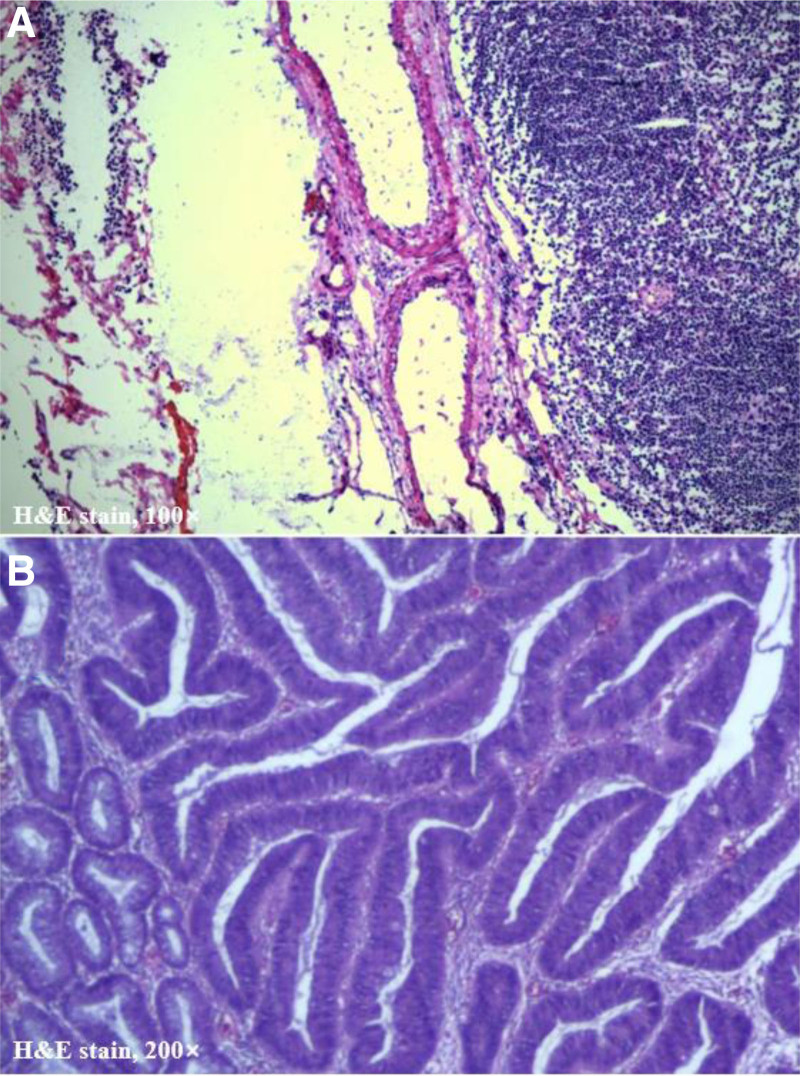
(A) Pathological examination showed one lymph node with reactive hyperplasia. (B) Postoperative pathology showed rectal tumor: tube-villous adenoma with high-grade intraepithelial neoplasia, focal canceration (intramucosal adenocarcinoma), cancer tissue did not invade the muscularis mucosa, and the resection margin was negative.

The patient underwent laparoscopic right LLND and pathological examination showed reactive hyperplasia of the lymph nodes (Fig. 3A). After ruling out the possibility that the enlarged lymph nodes were metastatic to rectal cancer, the patient received ESD treatment under general anesthesia. Postoperative pathology showed rectal tumor: tube-villous adenoma with high-grade intraepithelial neoplasia, focal canceration (intramucosal adenocarcinoma), cancer tissue did not invade the muscularis mucosa, and the resection margin was negative (Fig. 3B). The patient recovered well after surgery without complications and was discharged 12 days after surgery. The follow-up 3 months after surgery was good and the patient reported an excellent postoperative recovery due to the minimally invasive treatment. Written informed consent was obtained from the patient for the publication of this case report.

The sequence of clinical events is summarized in Table 1.

**Table 1:** Diagnostic-therapeutic timeline.

Milestone	Time point	Key findings/actions	Clinical significance
Symptom onset	March 24, 2024	Blood in stool for 1 mo	Initial presentation
Colonoscopy	April 25, 2024	Rectal mucosal mass and ulcerative colitis	Perform a further biopsy
Biopsy	April 25, 2024	Rectal villous adenoma with high-grade intraepithelial neoplasia, and focal canceration (intramucosal adenocarcinoma)	Early rectal malignant tumor (stage 0) was diagnosed
Pelvic MR and enhanced CT	May 4, 2024	Enlarged right-sided group 263D lymph nodes, with clear boundaries and a diameter of about 1.2 cm	The possibility of rectal cancer metastasis was considered.
Preoperative discussion	May 6, 2024	Decision: Diagnostic LLND prior to ESD	Rule out the possibility of metastasis.
LLND	May 7, 2024	Pathological examination showed one lymph node with reactive hyperplasia	Perform ESD resection of the tumor.
ESD	May 7, 2024	The tumor was completely resected with negative margins.	Complete resection of the tumor
Hospital discharge	May 19, 2024	No postoperative complications	Recovery milestone
Follow-ups	August 19, 2024	No residual or recurrent lesions	Treatment efficacy confirmation

CT = computed tomography, ESD = endoscopic mucosal dissection, LLND = lateral lymph node dissection, MR = magnetic resonance.

## 3. Discussion

The probability of lateral lymph node metastasis in ERC is 0.5% to 0.9%, which is extremely rare in clinical practice, so it is easy to ignore the occurrence of lateral lymph node metastasis when facing ERC patients.^[2]^ According to the CSCO Guidelines for Colorectal Cancer, endoscopic treatment is suitable for intramucosal cancer or superficial submucosal invasive cancer, especially ERC with submucosal infiltration <1mm, no lymphatic vessel invasion, well-differentiated tumors, no tumor budding, and the tumor is more than 1mm away from the incisal margin.^[4]^ ESD is the most suitable method for total resection of local lesions among endoscopic treatment methods, and it can completely remove the lesion in one go and the incisal margin is mostly negative, which can avoid damage caused by repeated surgical resection or total rectal resection. It is especially suitable for lesions with a size greater than half of the circumference of the rectal lumen and is one of the main treatment methods for ERC.^[3,5]^ Lateral lymph node metastasis is the most important cause of local recurrence of rectal cancer. When lateral lymph node enlargement exists, since conventional imaging examination cannot clarify the nature of the lymph node, LLND should be considered to clarify the nature of the lymph node while significantly reducing the local recurrence rate of rectal cancer.^[6]^ In this case, due to lateral lymph node enlargement, the nature of the lymph node was clarified through LLND, and the possibility of rectal cancer metastasis was ruled out before ESD resection was performed.

UC was first reported in 1859 and is one of the important risk factors for rectal cancer, especially when UC has a long course and a large spread.^[1]^ UC is closely related to changes and dysfunction of the lymph nodes, characterized by increased lymphangiogenesis, dilatation, and lymphadenopathy which can lead to reactive hyperplasia of lymph nodes and lymph node enlargement.^[7]^ This patient had UC combined with ERC and had enlarged lateral lymph nodes of unclear nature. Radical resection of rectal cancer combined with LLND could be considered. However, since UC would affect postoperative anastomotic healing,^[1]^ we hoped to find a more minimally invasive surgical method, so we performed LLND first to clarify the nature of the enlarged lymph nodes, and ESD was performed after clarifying that the enlarged lymph nodes was reactive hyperplasia. The patient recovered well postoperatively and no complications occurred, indicating that LLND combined with ESD is a safe and effective treatment strategy, which can not only make a clear diagnosis of enlarged lymph nodes but also completely remove the lesions.

In summary, the possibility of lymph node metastasis in ERC should not be overlooked. This case innovatively employed LLND combined with ESD to treat ERC with enlarged lateral lymph nodes of unknown nature. By first performing LLND to determine the nature of the lymph nodes, followed by ESD to completely resect the tumor, this strategy not only reduced the trauma to the patient but also alleviated the patient’s financial burden. This demonstrates that LLND combined with ESD has extremely high clinical application value. This case also emphasizes the importance of individualized diagnosis and treatment and the advantages of combined laparoscopic-endoscopic technique, providing reference for the diagnosis and treatment of similar cases.

## Author contributions

**Conceptualization:** RunZhi Liu.

**Writing – original draft:** Baosu Huang, Bing Zhu, RunZhi Liu.

**Writing – review & editing:** Baosu Huang, Bin Ren.
